# A bibliometric analysis of the frontiers and hotspots in chronic endometritis

**DOI:** 10.3389/fmed.2025.1559543

**Published:** 2025-05-16

**Authors:** Yunfang Luo, Qin Huang, Xing Li, Chaogang Yang, Jinli Ding

**Affiliations:** ^1^Reproductive Medical Center, Renmin Hospital of Wuhan University and Hubei Clinic Research Center for Assisted Reproductive Technology and Embryonic Development, Wuhan, China; ^2^Department of Obstetrics and Gynecology, Renmin Hospital of Wuhan University, Wuhan, China; ^3^Department of Gastrointestinal Surgery, Zhongnan Hospital of Wuhan University, Wuhan, China; ^4^Hubei Key Laboratory of Tumor Biological Behaviors, Wuhan, China; ^5^Hubei Cancer Clinical Study Center and the Clinical Medical Research Center of Peritoneal Cancer of Wuhan, Wuhan, China

**Keywords:** chronic endometritis, bibliometric analysis, infertility, pregnancy loss, CD138

## Abstract

**Background:**

Chronic endometritis (CE) is an inflammatory condition characterized by endometrial plasmacyte infiltration. The diagnosis and treatment of CE have attracted more and more attention. This article aimed to analyze the relevant keywords, development trends, and research hotspots of CE, which can provide a direction for future research.

**Materials and methods:**

The relevant articles on CE were retrieved from the Web of Science Core Collection. VOSviewer and CiteSpase were applied to analyze publication years, countries, institutions, journals, authors, citation and co-citation, co-occurrence and citation burst of keywords.

**Results:**

A total of 373 publications on CE were retrieved. Annual publication of articles about CE grew rapidly, and 2022 had the biggest outputs (50). USA contributed the most publications (87), and the University of Bari Aldo Moro had the highest number of articles in this field (27). The journal with the highest cited count and most publications was Fertility and Sterility (30). The main disciplines concerning the research on CE were Obstetrics Gynecology (141, 38.11%) and Reproductive Biology (126, 34.05%). Ettore Cicinelli, Kotaro Kitaya, and Dominique De Ziegler were the top three authors in publications. Except for CE and endometritis, the five most common keywords were infertility, hysteroscopy, plasma cell, recurrent implantation failure, and CD138. Pregnancy loss and recurrent miscarriage are currently within the burst period and might be the persistent research heated topics in this field.

**Conclusion:**

This study is useful for researchers to quickly grasp the current situation of CE research and enlighten researchers to explore new problems in CE.

## Introduction

Endometritis is an inflammatory disorder of the endometrium, which includes acute endometritis and chronic endometritis (CE). In contrast to acute endometritis, which presents with fever, pelvic pain, and vaginal discharge, the mild and undescribable symptoms of CE (pelvic discomfort, spotting, and leucorrhea) are often overlooked by patients and gynecologists (1). Acute endometritis is characterized by microabscess formation in the endometrium, not affecting pregnancy outcome and infertility (2), while CE is characterized by endometrial high stromal cell density and infiltration of endometrial stromal plasmacytes (3), which is closely associated with adverse pregnancy outcomes (4–8). Due to the lack of specific signs and symptoms, diagnosis of CE relies heavily on laboratory tests. For current clinical practice, histopathologic detection of endometrial stromal plasmacytes with marker CD138 (a typical molecular marker for plasma cells) in endometrial biopsy is reliable for the diagnosis of CE (9), but there are no technical standards and conditions for immunostaining of endometrial specimen regarding CD138 for now. In addition, hysteroscopy is a potential screening tool for CE (10), but hysteroscopy cannot replace histopathological examination with CD138 immunostaining for CE diagnosis (11, 12). Antibiotics have been proven to cure CE (13, 14), and antibiotic treatment can improve the pregnancy outcome for women with recurrent miscarriage (RM) and recurrent implantation failure (RIF) (15, 16).

Bibliometric analysis, a method to analyze literature in a field in order to do the statistical analysis and evaluation of literature, has received popularity in recent years. The relevant research on CE has developed rapidly, but there isn't any bibliometric analysis on CE. The aim of this study is to analyze and conclude the trends, hotspots, and important events in CE using the bibliometric analysis method, which may offer a new perspective on CE and have an important role in future research.

## Materials and methods

### Data collection and search strategy

**Figure 1:**
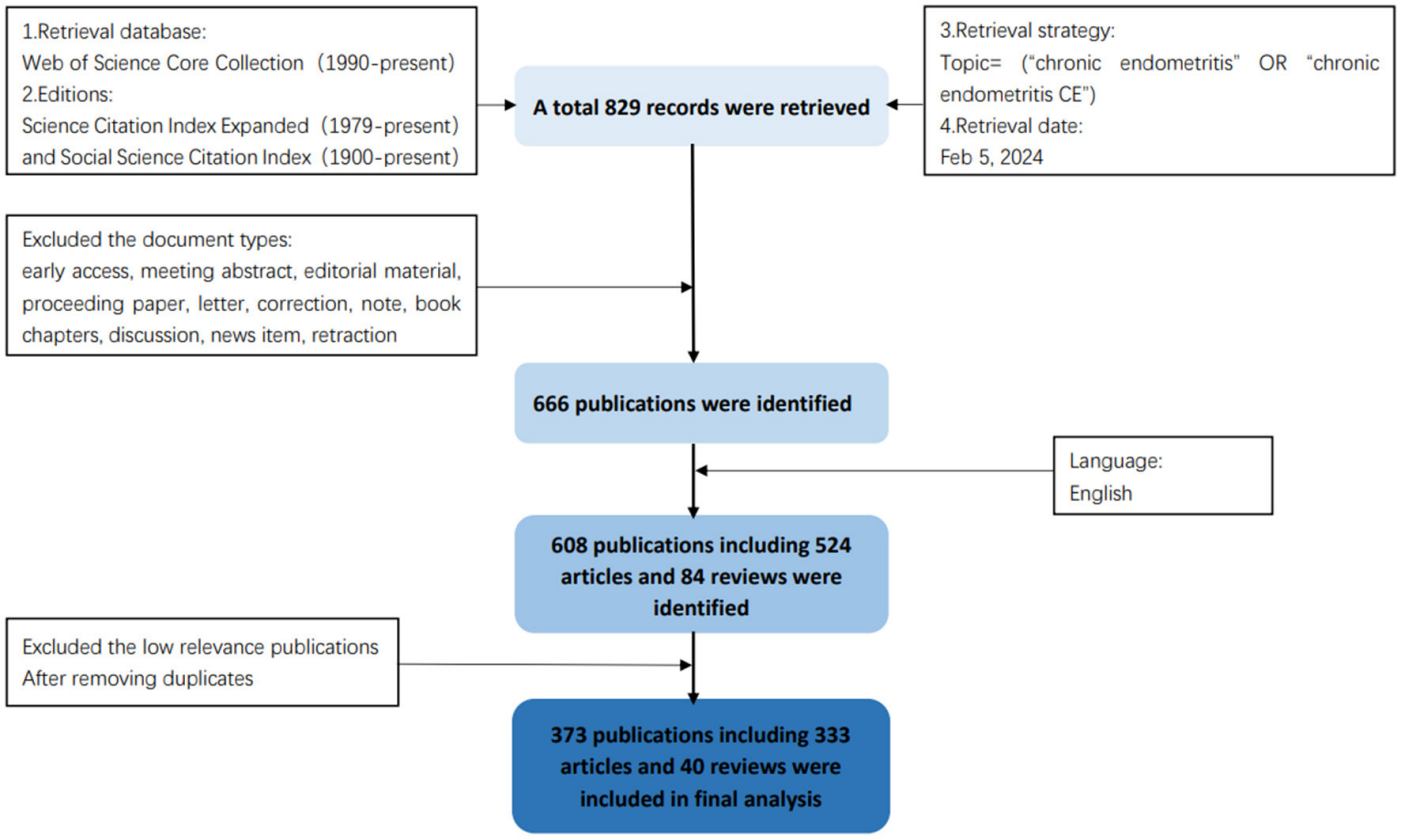
The mind map of the collection and origin of the data.

All the relevant articles on CE were retrieved from the Web of Science Core Collection (WOS), one of the most useful documents search databases. The details about data collection and origin were shown in Figure 1. To make the data more precise and reliable, we chose the Science Citation Index Expanded (1979-December 2023) and Social Science Citation Index (1900-December 2023) to retrieve literature. The search strategies were as follows: Topic = (“chronic endometritis” OR “CE”). As for the document types, only articles and reviews were included in the study. Publications only in English were included. There was a total of 608 publications. After excluding those unrelated publications, the remaining 373 publications were included in the subsequence analyses.

### Data extraction and analysis

The data retrieved from the Web of Science Core Collection were downloaded and exported into plain text file by choosing “Full Record and Cited References” for further analysis. After getting the data of annual publications and citations on CE, the result was represented in two histograms using Excel. CiteSpace and VOSviewer, two applications widely used for bibliometric analysis, were applied to further analyze the relationship and cooperation. CiteSpace (6.2.R6) was utilized to draw a visualized knowledge map by choosing author, country, institution and keywords as node types, respectively. The parameters of CiteSpace were set as follows: link retaining factor (LRF = 3.0), look back years (LBY = 5), time slicing (from 1994 to 2023), years per slice (2), selection criteria (g-index, *k* = 25). In the map, nodes represent various things such as authors, the size of nodes indicates the frequency, and the connections between nodes reflect the relation. Therefore, the top significant elements can be seen and distinguished from a mass of data. VOSviewer (1.6.20) was applied to visualize co-authorship among countries/organizations/authors, citation of documents and sources, co-citation of cited documents and co-occurrence of author keywords. The first step was to create a map based on retrieved data. Then different types were chosen to analyze, attaining various maps. Similarly, in the figures, the node embodies different types such as country, the size of the node shows the number of documents, the thickness of the line indicates the strength of the connection, and the color manifests the cluster.

## Results

### Temporal distribution map of the literature

**Figure 2:**
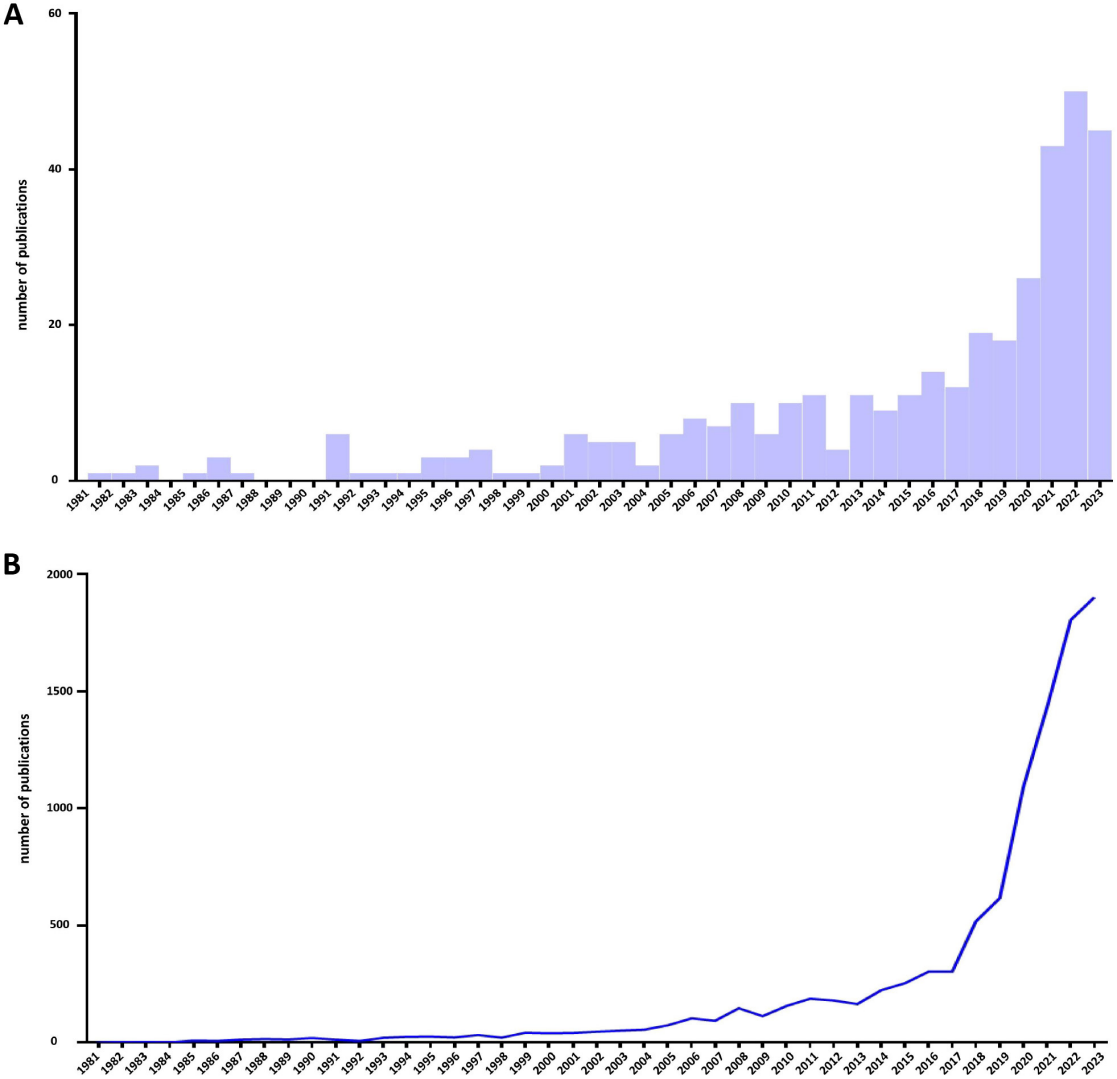
The publications and citations per year regarding chronic endometritis. **(A)** publications; **(B)** citations.

A total of 373 publications on CE were retrieved, with 40 reviews (10.72%) and 333 articles (89.28%), respectively. As shown in Figure 2A, the global trend of CE study was obvious and could be divided into three stages. From 1981 to 2000, CE emerged, and only had a few articles, therefore, this period can be identified as the initial stage. From 2000 to 2020, more publications in this field emerged, but the number of publications increased slowly, therefore, this period can be regarded as the developmental stage. Compared with the number of publications in 2020, the number in 2021 dramatically increased, which kept rising in 2022 and 2023, and the number of publications in 2022 reached a peak, indicating that CE has become a hot topic in recent years. As for the citations of the publications on CE, the trend was almost the same as the trend of publications, and the peak emerged in 2023 (Figure 2B).

### Distribution and cooperation of countries and institutions

**Figure 3:**
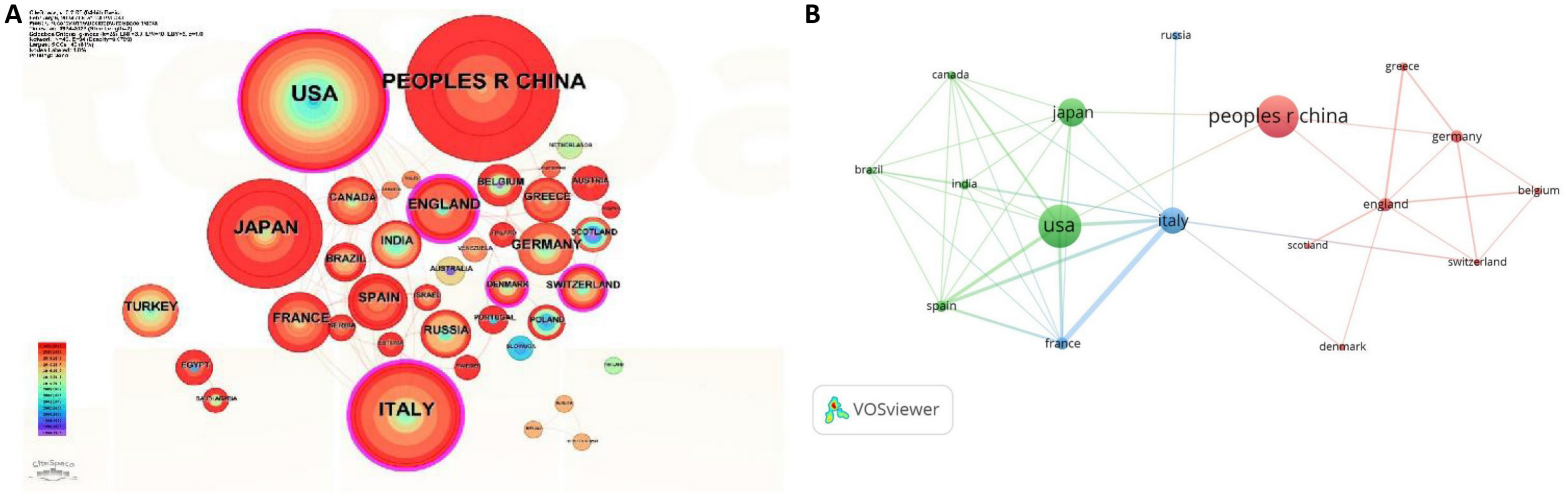
Countries contribution on chronic endometritis. **(A)** Countries contribution to the publications on chronic endometritis based on CiteSpace. **(B)** Network map of co-authorship between countries with more than five publications based on VOSviewer.

According to the research results from CiteSpace, a total of 173 countries/regions contributed to the publications in this field. As shown in Table 1, the USA contributed the greatest number of publications (87, 23.51%), followed by China (80, 21.62%), Japan (45, 12.16%), Italy (41, 11.08%), and England (17, 4.59%). Among them, the USA, Italy and England had relatively high centrality, which was represented by the pink ring surrounding the periphery of the circle of countries (Figure 3A). As shown in Figure 3A and Table 1, the study in this field in China and Japan has emerged and quickly developed in recent years, whereas the USA, Italy and England had a relatively long history. In addition, Italy (32), the USA (25), and England (11) were the top three countries for the total link strength, as the thickness of the line indicates the strength of the connection (Figure 3B).

**Table 1:** Top 5 countries in the production of chronic endometritis.

**Rank**	**Country**	**Region**	***Q***	**Percentage (%)**	**ACI**	***h***	**Total link strength**
1	USA	North America	87	23.51	39.79	35	25
2	China	Eastern Asia	80	21.62	17.64	20	4
3	Japan	Eastern Asia	45	12.16	30.56	17	9
4	Italy	Southern Europe	41	11.08	54.15	24	32
5	England	Western Europe	17	4.59	25.71	9	11

Q, quantity; ACI, average cited per item; h, h-index.

**Figure 4:**
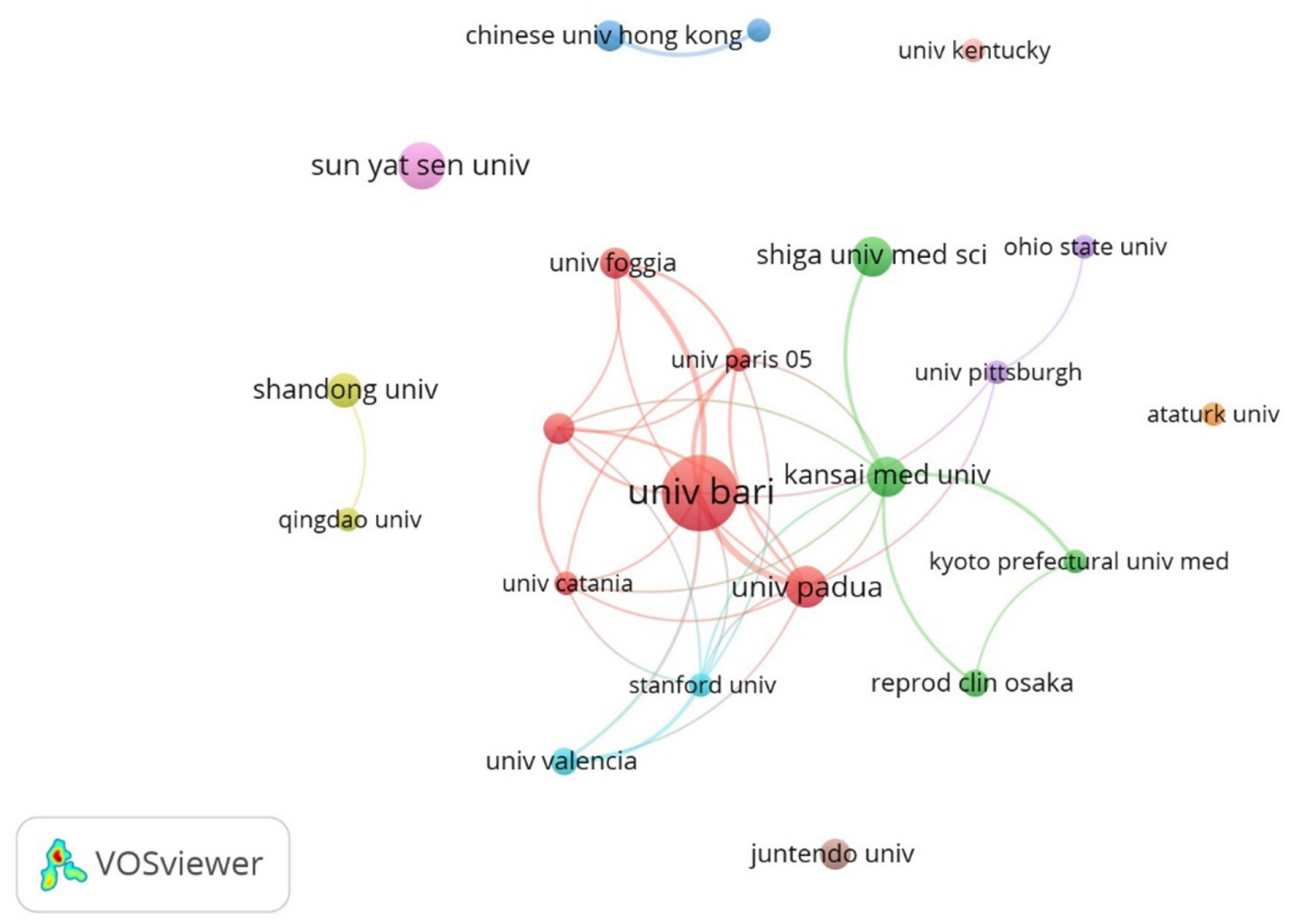
Network map of co-authorship between institutions with more than five publications.

As shown in Table 2 and Figure 4, the University of Bari Aldo Moro had the highest number of publications in this field, with a quantity of 27, followed by Sun Yat-sen University (13), University of Padua (11), Kansai Medical University (10), and Shiga University of Medical Science (10). Among these five top publication institutions, three institutions including the University of Bari Aldo Moro (total link strength = 26 times), the University of Padua (19), and Kansai Medical University (13) were the top three institutions for the total link strength.

**Table 2:** Top 5 institutions regarding the research on chronic endometritis.

**Rank**	**Institution**	**Country**	**Quantity**	**STC**	**ACI**	**Total link strength**
1	University of Bari Aldo Moro	Italy	27	1992	73.78	26
2	Sun Yat-sen University	China	13	313	24.08	0
3	University of Padua	Italy	11	523	47.55	19
4	Kansai Medical University	Japan	10	753	75.3	13
5	Shiga University of Medical Science	Japan	10	359	35.9	3

STC, sum of the times cited; ACI, average cited per item.

### Analysis of journals and disciplines

**Figure 5:**
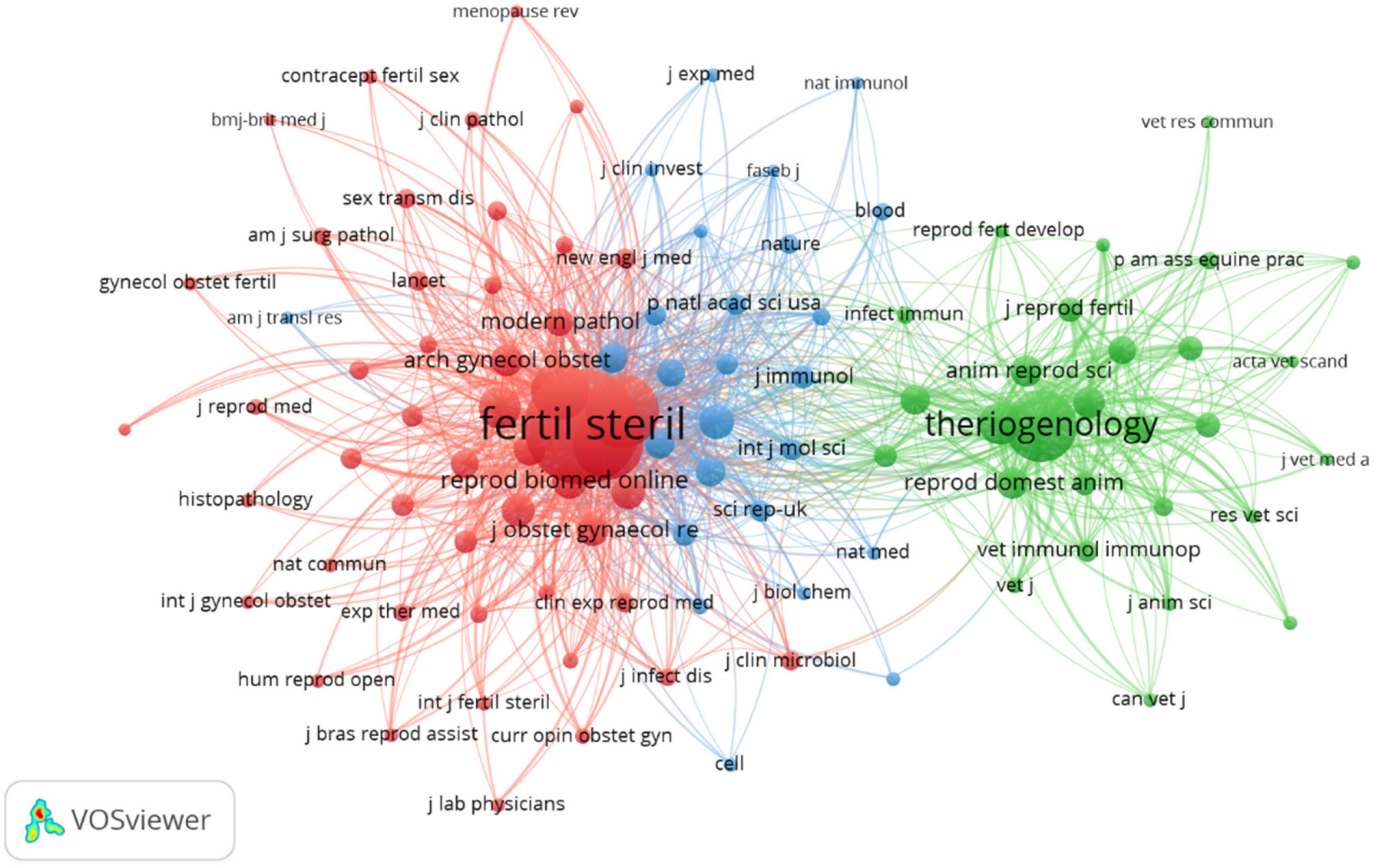
Network map of co-citation of the cited journals with more than 20 citations.

Based on the co-citation of the cited journals and published journals, we analyzed the core journals in this field. As shown in Table 3 and Figure 5, the journal with the highest number of publications in this field was Fertility and Sterility (30), followed by American Journal of Reproductive Immunology (25), Theriogenology (14), American Journal of Obstetrics and Gynecology (9), BMC Woman Health (9), and Journal of Obstetrics and Gynecology Research (9). The journal with the highest average cited per item (ACI) value was Fertility and Sterility (70.77), followed by American Journal of Obstetrics and Gynecology (63.67), Theriogenology (49.5), American Journal of Reproductive Immunology (38.56), and Journal of Minimally Invasive Gynecology (36.43). The top six journals in terms of h-index were Fertility and Sterility (23), American Journal of Reproductive Immunology (14), Theriogenology (11), American Journal of Obstetrics and Gynecology (9), Journal of Obstetrics and Gynecology Research (7), and Journal of Minimally Invasive Gynecology (7). The top three journals with high impact factors in 2023 were American Journal of Obstetrics and Gynecology (8.7), Fertility and Sterility (6.6), and Journal of Minimally Invasive Gynecology (3.5).

**Table 3:** Top 10 journals in the studies regarding chronic endometritis.

**Rank**	**Journal title**	**Quantity**	**ACI**	**2023IF**	***Q***	***h***
1	Fertility and sterility	30	70.77	6.6	Q1	23
2	American journal of reproductive immunology	25	38.56	2.5	Q3	14
3	Theriogenology	14	49.5	2.4	Q3	11
4	American journal of obstetrics and gynecology	9	63.67	8.7	Q1	9
5	BMC woman health	9	11.67	2.4	Q2	4
6	Journal of obstetrics and gynecology research	9	23.22	1.6	Q3	7
7	Journal of equine veterinary science	8	10	1.3	Q2	5
8	Diagnostics	7	9.14	3.0	Q1	5
9	Journal of assisted reproduction and genetics	7	17.14	3.2	Q2	6
10	Journal of minimally invasive gynecology	7	36.43	3.5	Q1	7

ACI, average citations per item; IF, impact factors; Q, quartile in the category; h, h-index.

As shown in Table 4, the top five subject categories with the highest publications were Obstetrics Gynecology (141, 38.11%), Reproductive Biology (126, 34.05%), Veterinary Sciences (92, 24.87%), Immunology (44, 11.89%), and Agriculture Dairy Animal Science (23, 6.22%).

**Table 4:** The top 10 subject categories concerning the research on chronic endometritis.

**Rank**	**WOS categories**	**Quantity**	**Percentage (%)**
1	Obstetrics gynecology	141	38.11
2	Reproductive biology	126	34.05
3	Veterinary sciences	92	24.87
4	Immunology	44	11.89
5	Agriculture dairy animal science	23	6.22
6	Medicine general internal	19	5.14
7	Pathology	19	5.14
8	Medicine research experimental	13	3.51
9	Public environmental occupational health	13	3.51
10	Microbiology	10	2.70

### Analysis of authors

**Figure 6:**
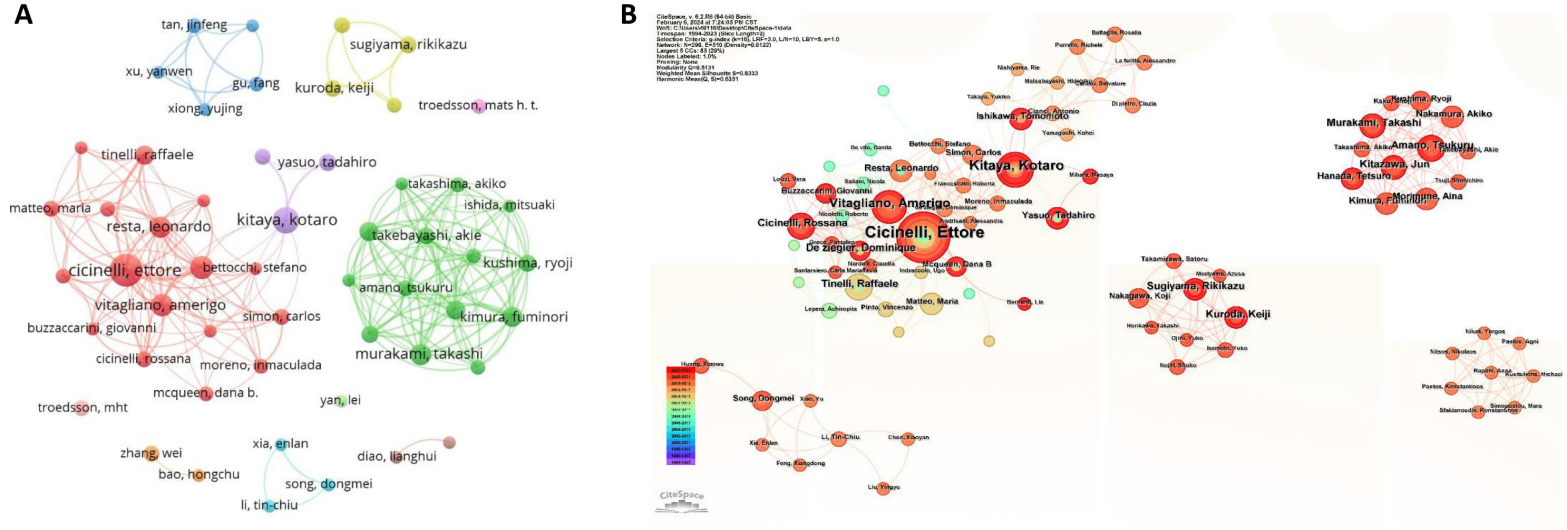
Co-authorship and cooperation between authors on chronic endometritis. **(A)** Network map of co-authorship between authors who published more than 4 publications based on VOSviewer. **(B)** Authors cooperation and their contribution to the publication based on CiteSpace.

Figure 6 displayed the distribution and cooperation of the authors. We analyzed a total of 53 authors that were co-authored in more than four publications. There were three main groups, the red one included Ettore Cicinelli, Dominique De Ziegler, Amerigo Vitagliano, Leonardo Resta, and Raffaele Tinelli; while the green one included Takashi Murakami, Fuminori Kimura, Jun Kitazawa, and Akiko Nakamura; the purple one included Kotaro Kitaya, who cooperated with other authors. The five authors with the highest total link strength were Ettore Cicinelli (72), Takashi Murakami (67), Fuminori Kimura (62), Jun Kitazawa (61), and Akiko Nakamura (61).

As shown in Table 5, Ettore Cicinelli from the University of Bari Aldo Moro had the highest number of published articles (26), followed by Kotaro Kitaya from Kansai Medical University (16) and Dominique De ziegler from Paris-Saclay University (13). The top three authors in terms of ACI values were Raffaele Tinelli (101.88), Leonardo Resta (89.75), and Ettore Cicinelli (76.31).

**Table 5:** Top 12 productive authors in the studies of chronic endometritis.

**Rank**	**Author**	**Country**	**Institution**	**TP**	***P* (%)**	**ACI**	***h***
1	Cicinelli, Ettore	Italy	University of Bari Aldo Moro	26	7.03	76.31	20
2	Kitaya, Kotaro	Japan	Kansai Medical University	16	4.32	50.94	10
3	De Ziegler, Dominique	France	Paris-Saclay University	13	3.51	65	12
4	Resta, Leonardo	Italy	University of Bari Aldo Moro	12	3.24	89.75	12
5	Troedsson, Mats H. T.	United States	University of Kentucky	11	2.97	47.18	7
6	Vitagliano, Amerigo	Italy	University of Bari Aldo Moro	11	2.97	47.55	10
7	Murakami, Takashi	Japan	Shiga University of Medical Science	10	2.70	35.90	8
8	Kimura, Fuminori	Japan	Nara Medical University	9	2.43	38.67	7
9	Kuroda, Keiji	Japan	Kitasato University	8	2.16	11.25	5
10	Kitazawa, Jun	Japan	Shiga University of Medical Science	8	2.16	24.75	7
11	Nakamura, Akiko	Japan	Aichi Medical University	8	2.16	24.75	7
12	Tinelli, Raffaele	Italy	University of Bari Aldo Moro	8	2.16	101.88	8

TP, total publications; P, percentage; ACI, average citations per item; h, h-index.

### Citation and co-citation analyses

The top 10 documents with the highest citations were shown in Table 6. There were 304 citations for “Recurrent implantation failure-update overview on etiology, diagnosis, treatment and future directions” (5), followed by “Prevalence of chronic endometritis in repeated unexplained implantation failure and the IVF success rate after antibiotic therapy” (17), with 268 citations. The third-ranked article with the largest number of citations was “Chronic endometritis is a frequent finding in women with recurrent implantation failure after *in vitro* fertilization” (6), with 233 citations.

**Table 6:** Top 10 citation analysis of documents regarding chronic endometritis.

**Rank**	**Title**	**Journal**	**Type**	**Authors**	**Y**	**C**	**R**
1	Recurrent implantation failure-update overview on etiology, diagnosis, treatment and future directions	Reproductive biology and endocrinology	Review	Bashiri, Asher	2018	304	112
2	Prevalence of chronic endometritis in repeated unexplained implantation failure and the IVF success rate after antibiotic therapy	Human reproduction	Article	Cicinelli, Ettore	2015	268	32
3	Chronic endometritis is a frequent finding in women with recurrent implantation failure after *in vitro* fertilization	Fertility and sterility	Article	Johnston-MacAnanny, Erika B	2010	233	25
4	Bovine haptoglobin response in clinically defined field conditions	Veterinary record	Article	Skinner, JG	1991	217	16
5	Chronic endometritis due to common bacteria is prevalent in women with recurrent miscarriage as confirmed by improved pregnancy outcome after antibiotic treatment	Reproductive sciences	Article	Cicinelli, Ettore	2014	174	35
6	Chronic endometritis in women with recurrent pregnancy loss and recurrent implantation failure: prevalence and role of office hysteroscopy and immunohistochemistry in diagnosis	Fertility and sterility	Article	Bouet, Pierre-Emmanuel	2016	162	26
7	Influence of puerperal uterine infection on uterine involution and postpartum ovarian activity in dairy cows	Reproduction in domestic animals	Article	Mateus, L	2002	158	55
8	The diagnosis of chronic endometritis in infertile asymptomatic women: a comparative study of histology, microbial cultures, hysteroscopy, and molecular microbiology	American of obstetrics and gynecology	Article	Moreno, Inmaculada	2018	153	47
9	Chronic endometritis—morphologic and clinical observations	Obstetrics and gynecology	Article	Greenwood, SM	1981	152	20
10	Uterine clearance and resistance to persistent endometritis in the mare	Theriogenology	Article	Troedsson, MHT	1999	140	75

Y, publication year; C, citations; R, references.

Table 7 listed the top 10 cited references with the highest co-citations. The five references with the largest number of citations were by Cicinelli, Ettore (17), Johnston-MacAnanny, Eeika B (6), Bouet, Pierre-Emmanuel (18), Greenwood, SM (19), and Kitaya, Kotaro (20).

**Table 7:** Top 10 co-citation analysis of cited reference on chronic endometritis.

**Rank**	**Title**	**Journal**	**Type**	**Authors**	**Y**	**C**	**R**
1	Prevalence of chronic endometritis in repeated unexplained implantation failure and the IVF success rate after antibiotic therapy	Human reproduction	Article	Cicinelli, Ettore	2015	268	32
2	Chronic endometritis is a frequent finding in women with recurrent implantation failure after *in vitro* fertilization	Fertility and sterility	Article	Johnston-MacAnanny, Erika B	2010	233	25
3	Chronic endometritis in women with recurrent pregnancy loss and recurrent implantation failure: prevalence and role of office hysteroscopy and immunohistochemistry in diagnosis	Fertility and sterility	Article	Bouet, Pierre-Emmanuel	2016	162	26
4	Chronic endometritis—morphologic and clinical observations	Obstetrics and gynecology	Article	Greenwood, SM	1981	152	20
5	Live birth rate following oral antibiotic treatment for chronic endometritis in infertile women with repeated implantation failure	American journal of reproductive immunology	Article	Kitaya, Kotaro	2017	138	28
6	Chronic endometritis due to common bacteria is prevalent in women with recurrent miscarriage as confirmed by improved pregnancy outcome after antibiotic treatment	Reproductive sciences	Article	Cicinelli, Ettore	2014	174	35
7	Chronic endometritis: correlation among hysteroscopic, histologic, and bacteriologic findings in a prospective trial with 2190 consecutive office hysteroscopies.	Fertility and sterility	Article	Cicinelli, Ettore	2008	144	27
8	The impact of chronic endometritis on reproductive outcome	Fertility and sterility	Article	Kasius, Jenneke C	2012	110	23
9	Pregnancy outcomes in women with chronic endometritis and recurrent pregnancy loss	Fertility and sterility	Article	McQueen, Dana B	2015	129	15
10	Chronic endometritis in women with recurrent early pregnancy loss and/or fetal demise	Fertility and sterility	Article	McQueen, Dana B	2014	125	14

Y, publication year; C, citations; R, references.

### Research hotspots and frontier analysis

**Figure 7:**
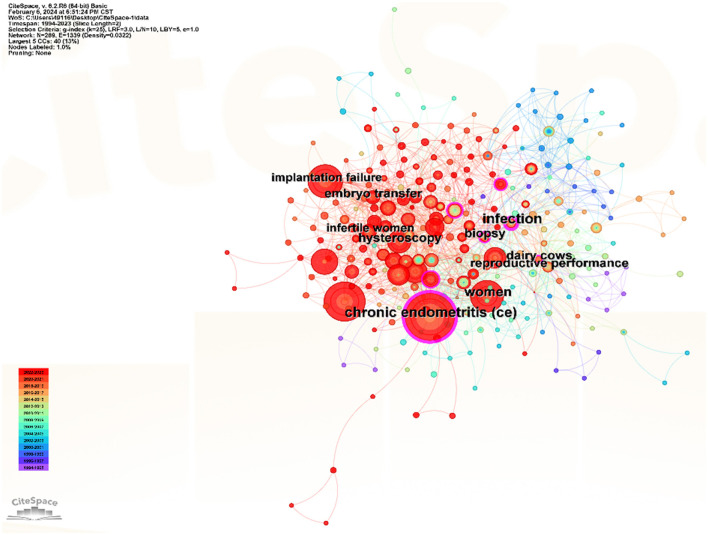
The distribution of keywords based on CiteSpace.

**Figure 8:**
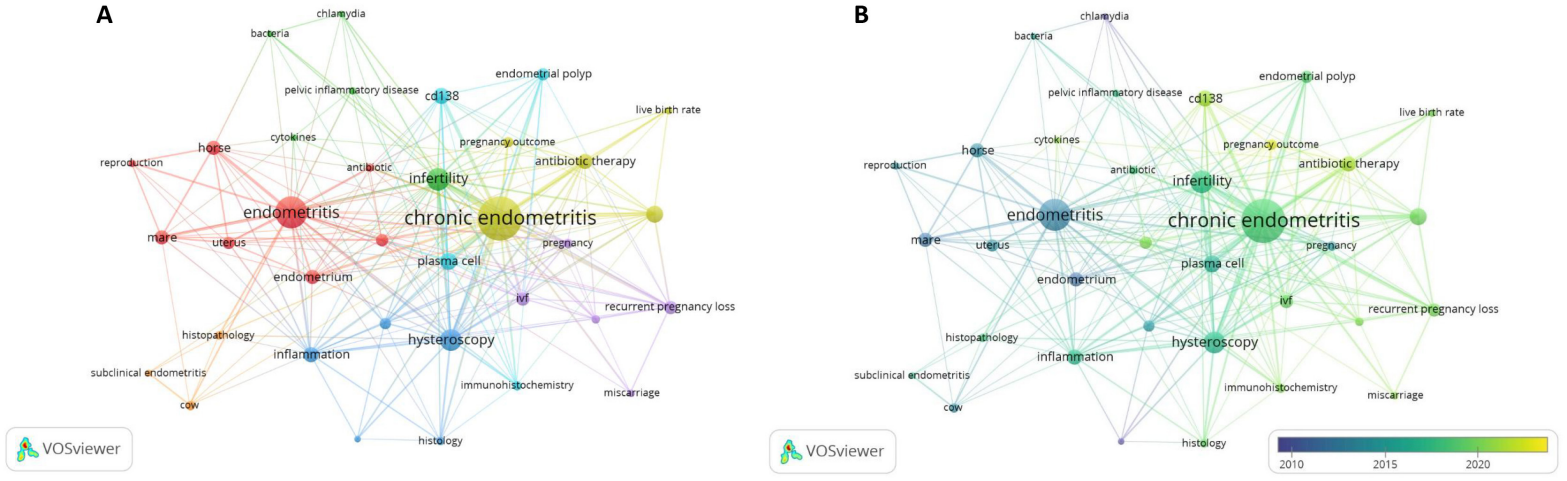
Network visualization of keywords. **(A)** Network visualization of keywords occur more than five times in research on chronic endometritis based on VOSviewer. **(B)** Network visualization of keywords occur more than five times with timeline based on VOSviewer.

Keywords are vitally significant for the analysis of the hotspots in one field. Through analyzing those keywords, researchers may find the frontier of the field, which is beneficial to the development of the subsequent research. By using CiteSpace and VOSviewer, we gained insight into the research and heat topic in the field of CE. Figure 7 displayed the keywords in CE generally. The main keywords included chronic endometritis, implantation failure, embryo transfer, hysteroscopy, infection, biopsy, dairy cows, reproductive performance, and woman. Table 8 showed the top 20 keywords in this field. In addition to chronic endometritis, keywords with a high frequency of occurrence were endometritis (95), infertility (51), hysteroscopy (45), plasma cell (29), recurrent implantation failure (29), and CD138 (24), which indicated the top hot keywords in CE. By using VOSviewer, we got a more detailed result by selecting the occurring time. A total of 35 keywords which occurred more than five times were analyzed. The size of the node indicated the occurrences, and the thickness of the lines between the two nodes represented the co-occurrence frequence (Figure 8A). What's more, the keywords formed seven clusters, which represented the major research directions in the field. The red cluster focused on the endometritis, endometriosis, endometrium, antibiotic, horse, mare, reproduction, and uterus. The green cluster included bacteria, chlamydia, cytokines, infertility, and pelvic inflammatory disease. The keywords in the dark blue cluster were endometrial biopsy, endometrial micropolyps, histology, hysteroscopy, and inflammation, which mainly demonstrated the diagnosis technique of CE. The yellow cluster was composed of antibiotic therapy, chronic endometritis, live birth rate, pregnancy outcome, and recurrent implantation failure, which mainly focused on the therapy and related factors of CE. The keywords in the purple cluster were implantation failure, IVF, miscarriage, pregnancy, and recurrent pregnancy loss, which placed the key point to the relevant important events caused by or associated with CE. The light blue cluster was made up of CD138, endometrial polyp, immunohistochemistry, and plasma cell. The orange cluster included cow, histopathology, and subclinical endometritis. Figure 8B displayed the first year of occurrence of keywords. Pregnancy outcome was thought to be the new star in the area of keywords.

**Table 8:** Top 20 keywords on chronic endometritis.

**Rank**	**Keywords**	**Occurrences**	**Total link strength**
1	Chronic Endometritis	189	305
2	Endometritis	95	128
3	Infertility	51	124
4	Hysteroscopy	45	115
5	Plasma cell	29	58
6	Recurrent implantation failure	29	57
7	CD138	24	46
8	Antibiotic therapy	23	57
9	Inflammation	22	52
10	Endometrium	21	35
11	Mare	20	39
12	Horse	19	39
13	IVF	17	48
14	Recurrent pregnancy loss	17	43
15	Endometriosis	15	31
16	Endometrial polyp	14	31
17	Uterus	14	28
18	Endometrial biospy	12	32
19	Pregnancy outcome	10	23
20	Pregnancy	9	26

**Figure 9:**
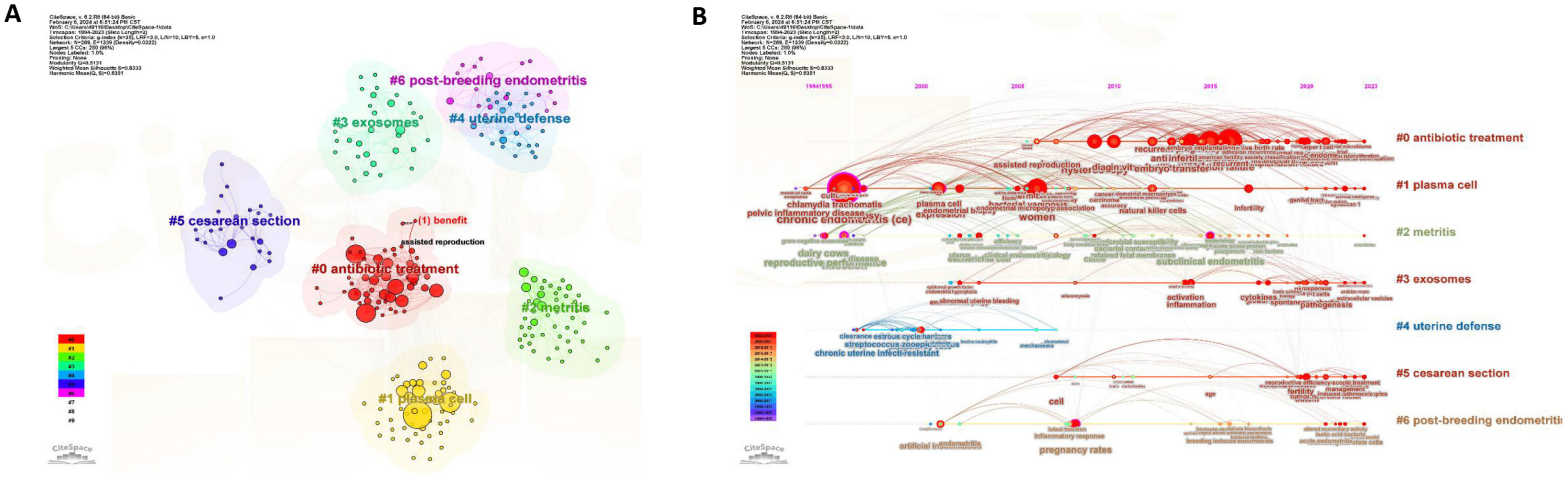
The cluster and timeline of keywords on chronic endometritis. **(A)** The cluster of keywords based on CiteSpace. **(B)** The timeline of keywords according to the cluster.

**Figure 10:**
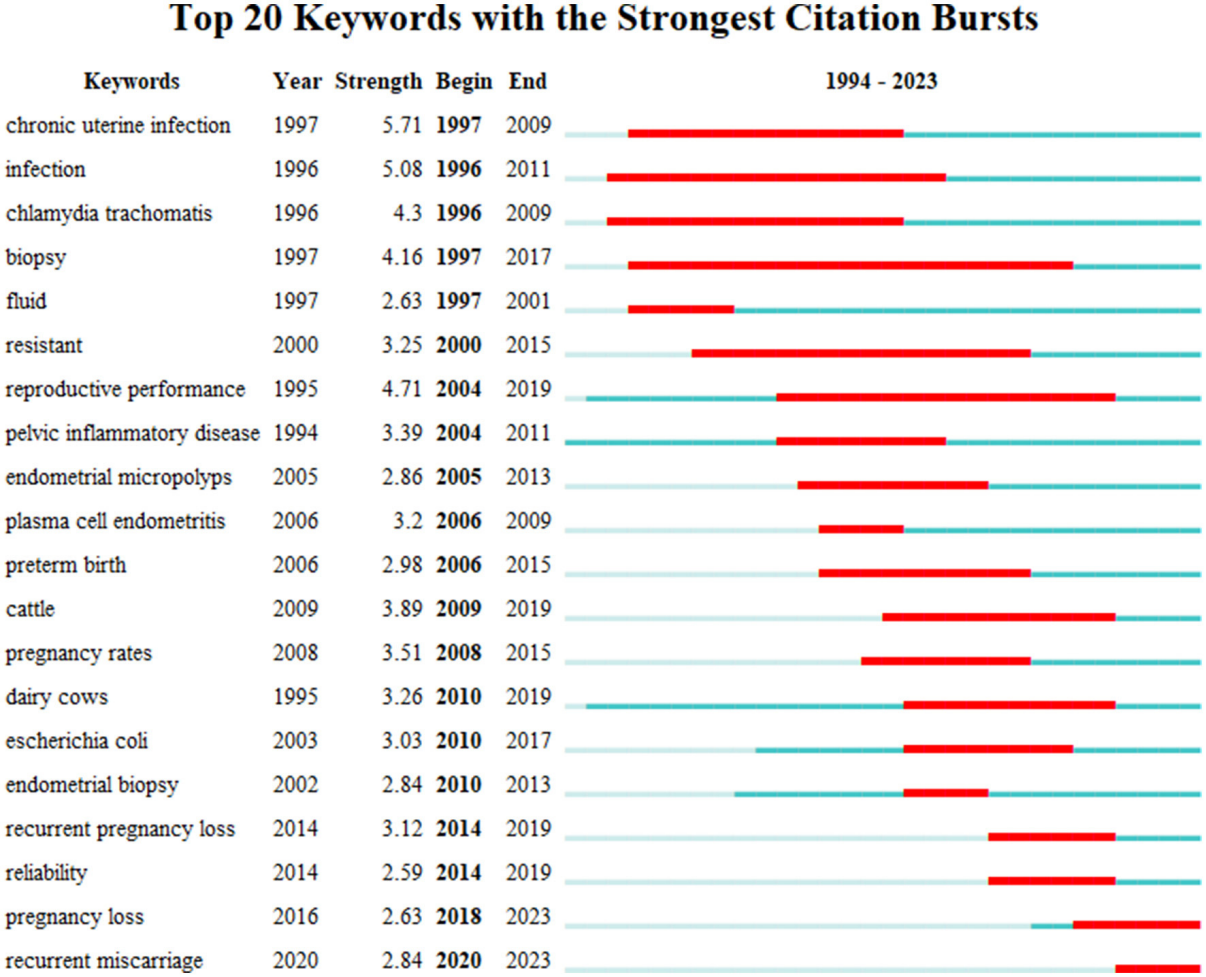
Top 20 keywords with the strongest citation bursts based on CiteSpace.

As displayed in Figure 9A, the keywords were divided into seven clusters. They were antibiotic treatment, plasma cell, metritis, exosomes, uterine defense, cesarean section, and post-breeding endometritis. Through the use of the CiteSpace, we analyzed the timeline of the keywords, which were classified according to the keywords. The holistic developmental trend was presented in Figure 9B. There were seven clusters. The important points in the cluster of antibiotic treatment were live birth rate, recurrent pregnancy loss, and repeated implantation failure. The vital points in the cluster of plasma cell included *chlamydia trachomatis*, pelvic inflammatory disease, plasma cell, expression, bacterial vaginosis, and preterm birth, which may be the hottest cluster in the seven clusters. The significant points in the cluster of metritis were dairy cows, reproductive performance, *Escherichia coli*, cattle, and subclinical endometritis. Abnormal uterine bleeding, activation, inflammation, cytokines, pathogenesis, and NK cells were the key points in the cluster of exosomes. Pregnancy rate, chronic uterine infection, and resistant were essential points in the cluster of uterine defense. The main key points in the cluster of cesarean section were fertility, reproductive efficiency, B cell, age, and cesarean-induced isthmocele. Endometritis, pregnancy rate, inflammatory response, antibiotic penetration, breeding induced endometritis, lactic acid bacteria and stem cells were the significant points in the cluster of post-breeding endometritis. Moreover, to further analyze the significant events and the trend in the future, we analyzed the burst pattern of keywords. As shown in Figure 10, the blue line represented the year in which the keywords didn't burst out, and the red line was on behalf of the burst time span. Chronic uterine infection ranked first with the highest burst strength (5.71), followed by infection (5.08), reproductive performance (4.71), *chlamydia trachomatis* (4.30), and biopsy (4.16). Pregnancy loss and RM are currently within the burst period and these two topics might be the persistent research heated topics in this field.

## Discussion

In this study, CiteSpace and VOSviewer were used to analyze the 373 CE-related publications published from 1981 to 2023. We analyzed the annual publications, citations trends, journals, authors, institutions, countries, and keywords. The study aims to provide a reference for CE researchers.

From 2020, the number of global CE publications displays an increasing trend, and the number of CE-related articles was predicted to increase durably in the future based on the current growth trend. Our results showed that Fertility and Sterility has the highest number of publications, American Journal of Reproductive Immunology, Theriogenology, American Journal of Obstetrics and Gynecology, BMC Woman Health, and Journal of Obstetrics and Gynecology Research were the core journals. In the future, advances and frontiers in CE may be also published in these journals. Through the h-index, we found that Ettore Cicinelli, De Ziegler Dominique, and Resta, Leonardo were the leaders in CE field. As for the academic institution, University of Bari Aldo Moro in Italy, Sun Yat-sen University in China, and University of Padua in Italy published the highest number of articles on CE. The USA contributed the highest number of CE-related articles, six of the top 12 authors were from Japan, and four of them were from Italy, which verified the outstanding contribution of these countries. Therefore, these researchers, institutions, and countries are likely to continue to play the leading role in CE research in the future.

Through the analysis of keywords, we found that infertility, hysteroscopy and plasma cell were the focus of CE research in the past decade. A high prevalence of CE was described in infertility (17), and the prevalence of CE in infertile women was higher than that observed in fertile ones (21) and general population (22). The associations between CE and fallopian tubal obstruction (9, 23, 24), endometriosis (23, 25) have been reported, which were the main sources of infertility. The pathophysiological link of CE on infertility has been demonstrated in depth. It is widely acknowledged that the etiology of CE is tightly connected with microbial infection in the endometrial cavity (16), which could induce aberrant expression of chemokines and adhesion molecules in the endometrium, leading to the migration of B cells to the endometrium, which might disturb the expression of genes associated with endometrial receptivity (26, 27). In addition, the expression of E-selectin could be induced by bacterial lipopolysaccharides, which increased the expression of CXCL13 and CXCL1, activated B-cell adhesion molecules in the endometrium, leading to an abnormal immune response in the stroma (28). The expression of immunoglobulins in the stromal plasma cells might have an adverse effect on embryo implantation (29). Hysteroscopy is an important tool that realizes the real-time visualization of the uterine cavity. The diagnostic criteria for CE were proposed by the International Working Group for the Standardization of Chronic Endometritis Diagnosis in 2019, which included strawberry aspect, focal hyperemia, endometrial micropolyps and stromal edema (30). However, Song et al. reported that the overall diagnosis accuracy of hysteroscopy for CE was 67% (11), indicating that hysteroscopy could not replace the histologic examination for the diagnosis of CE. CD138, also known as syndecan-1, is expressed on the plasmacytes. It has been demonstrated that immunohistochemistry for CD138 markedly improved the sensitivity and specificity in the histopathologic diagnosis of CE, which is the most preferred method for CE diagnosis. However, there are several limitations to its clinical utilization, including the specificity of CD138 expression in the endometrium, lack of standardized techniques and conditions, sampling method and device used in biopsy, and cutoff value for plasmocyte density.

Notably, the function of NK cells in CE has also drawn researchers' interest. It has been reported that there was a significant correlation between high uNK cell density and CE in women with RM (31, 32), and one study indicated that only immunoactive NK2 and cytotoxic NK4 subgroups were significantly increased in women in the RM or RIF groups (33). However, another study has shown that uNK cell does not change significantly between non-CE and CE patients with RM or RIF (34). Furthermore, women carrying the KIR AA genotype exhibit significantly lower levels of TNF-α and IL-1L in the uterus, which may increase susceptibility to chronic endometritis (35).

The future hot spot of CE may be closely related to pregnancy loss and RM. A higher incidence of CE has been reported in women with RM (36, 37). In addition, the live birth rate for women with RM and untreated CE was only 7% (37). What's more, antibiotic treatment could significantly improve the ongoing pregnancy rate (4, 20, 37). Additionally, studies have reported associations between CE and other pregnancy complications. CE may lead to sustained activation of the maternal immune system, resulting in abnormal placentation or vascular dysfunction, thereby increasing the risk of preeclampsia (38, 39). The endometrial inflammation caused by CE could extend to the placenta, inducing chronic deciduitis (40), which compromises placental barrier function and may trigger preterm labor (41). Notably, antibiotic-treated CE patients demonstrate comparable risks of preterm birth and preeclampsia to those without CE history (42, 43).

Currently, the term CE is widely used to describe chronic inflammatory conditions of the endometrium. However, research indicates that its pathogenesis is highly heterogeneous, involving both infectious factors and non-infectious factors. Notably, antibiotic therapy is only effective in a subset of CE patients, highlighting the complexity of its pathophysiological mechanisms (44). Recent studies suggest that, given the endometrium's inherent mild inflammatory state under physiological conditions and the diverse pathogenesis of CE, this condition should be more accurately redefined as “impaired inflammatory state of the endometrium” (IISE) (45). Research has found that IISE is closely associated with various non-infectious factors, such as metabolic abnormalities, hormonal fluctuations, and psychological stress, while also demonstrating significant links with conditions like endometrial polyps and gynecological malignancies (45). Based on these findings, researchers have proposed an innovative treatment strategy-“minimal effective dose pro-inflammatory therapy”, which aims to precisely modulate the endometrial inflammatory microenvironment to restore normal function (45). This approach offers a novel perspective for managing CE/IISE and may represent an important direction for future clinical interventions.

This study was the first bibliometric analysis of CE. However, it had some limitations. First, we only chose WOS to retrieve data, so there might be some publications that hadn't been recruited. Second, we only included publications in English, which would lead to the miss of some findings written in other languages. Finally, some articles published recently were not cited heavily, resulting in the possible omission of the hotspot analyses.

## Conclusion

In summary, bibliometrics results demonstrate that the number of CE articles from 2000 to 2023 increased yearly. Fertility and Sterility is prolific for the number of publications and h-index. University of Bari Aldo Moro in Italy is the most outstanding institution, and USA is the country with the largest number of publications. Ettore Cicinelli, Kotaro Kitaya and Dominique De Ziegler are the research leaders in CE field. Researches on CE have focused on infertility, hysteroscopy and plasma cell in the past decade. Pregnancy loss might be the hot spot in the future.

## Data Availability

The original contributions presented in the study are included in the article/supplementary material, further inquiries can be directed to the corresponding authors.
